# Assessment of Lipid Quality in Commercial Omega-3 Supplements Sold in the French Market

**DOI:** 10.3390/biom12101361

**Published:** 2022-09-23

**Authors:** Federica Pasini, Ana Maria Gómez-Caravaca, Thierry Blasco, Jelena Cvejić, Maria Fiorenza Caboni, Vito Verardo

**Affiliations:** 1 Department of Agricultural and Food Sciences, University of Bologna, Piazza Goidanich 60, 47521 Cesena, Italy; 2 Inter-Departmental Centre for Agri-Food Industrial Research (CIRI Agroalimentare), University of Bologna, Via Quinto Bucci 336, 47521 Cesena, Italy; 3 Department of Analytical Chemistry, Faculty of Sciences, University of Granada, Avd. Fuentenueva s/n, 18071 Granada, Spain; 4 Institute of Nutrition and Food Technology ‘José Mataix’, Biomedical Research Center, University of Granada, Avda del Conocimiento sn., 18100 Armilla, Spain; 5 INSU-CNRS, Laboratoire d’Océanographie de Villefranche, Sorbonne Université, 06230 Villefranche-sur-Mer, France; 6 Department of Pharmacy, Faculty of Medicine, University of Novi Sad, 21000 Novi Sad, Serbia; 7 Department of Nutrition and Food Science, University of Granada, Campus of Cartuja, 18071 Granada, Spain

**Keywords:** polyunsaturated fatty acids, lipid oxidation, fish oil, microalgae oil, *Schizochytrium*, peroxide value, *p*-anisidine value, Totox

## Abstract

Supplementation of omega-3 fatty acids is considered a valuable strategy to supply the low intake of these fatty acids. Thus, the safety of the supplements is an important milestone. Because of that, we analyzed 20 unflavored supplements sold in the French market for fatty acid and triglyceride composition, for EPA and DHA, and for tocol content, as well as for oxidative status. This study found that only 2.5% of the supplements did not meet their label claims for omega-3 content. TAG analysis showed high variability among the triglyceride distribution, and the same trend was also noticed for the tocol content; in fact, a high variability of the distribution of the six tocols (four tocopherols and two tocotrienols) was found among the samples. Of the tested products, all of them complied with peroxide value, *p*-anisidine value, and Totox values established by the Global Organization for EPA and DHA Omega-3s (GOED) and were not oxidized.

## 1. Introduction

Omega-3 fatty acids are distributed in different foods and are responsible for several health benefits. Despite the fact that alpha-linolenic acid (ALA) is the omega-3 fatty acid universally available in animal and plant sources, EPA and DHA present in marine sources are more bioactive and they are responsible of the main health benefits [1,2].

A relation between the high intake of EPA and DHA and a low risk of developing cardiovascular diseases (CVD) was demonstrated; this is due to the ability of these fatty acids to modulate several risk factors associated to CVD such as blood pressure, platelet aggregation, blood lipids, and inflammation [3]. According to Giacobbe et al. [4], omega-3 fatty acids reduce the symptoms of depression and have anti-inflammatory activity thanks to the formation of derived metabolites promoting them for tailored therapy in psychiatric, neurodegenerative, and neurological conditions. Moreover, DHA is also involved in the brain development processes and is important in patients with Alzheimer’s disease [5].

Another study from Mozaffari and co-workers [6] demonstrated an inverse correlation between omega-3 fatty acids and Crohn’s disease and ulcerative colitis.

A review of Rondanelli et al. [7] underlined the importance of an adequate intake of omega-3 fatty acids in order to maintain muscle mass and prevent the sarcopenia; in fact, these compounds reduce inflammation status thanks to the improvement of muscle response to exercise and diet.

Briefly, omega-3 fatty acids lead to reduction in the blood pressure and they are biologically active, inducing apoptotic cell death in cancer cells and exerting anti-cancer activity in vitro [8]. At the same time, they are considered immunonutrients and are used during cancer therapy [9].

Due to these benefits associated to the intake of omega-3 fatty acids, the World Health Organization (WHO) and the Food and Agriculture Organization (FAO) recommended an intake of 0.25–2 g EPA + DHA per day [10]. The European Food Safety Authority (EFSA) set a daily intake of 250 mg of long-chain omega-3 fatty acids for adults in order to reduce the risk of heart disease [11].

In spite of these data, Sioen and co-workers [12] have recently noticed that three-quarters of the countries considered in their study did not meet the EFSA recommendations in terms of intake of EPA + DHA. These data agreed with the results presented by Stark et al. [13], observing low blood levels of EPA and DHA in America (North, Central, and South), Europe, the Middle East, Southeast Asia, and Africa.

Thus, to reach the recommended intake, it is important to supplement omega-3 intake. In this way, the market offer of omega-3 supplements [14] or fortified foods is very high.

The sources of omega-3 oil for supplements are fish, krill, and microalgae. Fish oil is obtained from fish or fish by-products using a wet extraction followed by a refining process. Krill oil is rich in phospholipids, and microalgae oil is obtained after a more long and expensive process compared to fish oil, but it is a valuable choice for vegetarian people [15].

As expected, the fatty acid composition of the different supplement depends on several factors such as the oil source, the raw material used for the oil extraction, and the processes used to obtain the oil, among others. In addition, omega-3 fatty acids are susceptible to lipid oxidation and the presence of natural antioxidants, and the process of encapsulation is very important to ensure the safety of the final product. Because of that, the aim of this work is to evaluate the triglyceride, fatty acid, tocopherol, and tocotrienol composition, as well as the lipid oxidation status of omega-3 supplements in the French market.

## 2. Materials and Methods

### 2.1. Chemicals and Reagents

All the reagents were purchased from Merck (Darmstadt, Germany). GLC-463 mix was from Nu-Check (Elysian, MN, USA), and EPA, DHA, FAME 189-19, menhaden oil, pure triglycerides, and (+)-α-tocopherol were from Sigma-Aldrich (St. Louis, MO, USA).

### 2.2. Samples

A total of 20 omega-3 supplements without flavoring from different brands were purchased from the French market (supplement shops, parapharmacies, and pharmacies). Three bottles from the same batch were purchased for each supplement and they were stored at 4 °C until required for the analyses.

### 2.3. Fatty Acid Determination by GC FID

Fatty acids were determined as methyl-esters (FAMEs) according to Verardo et al. [16] by using a gas chromatograph from Shimadzu (GC-2010 Plus, Shimadzu Corporation, Kyoto, Japan) equipped with a flame ionization detector (FID); separation was carried out on a fused silica capillary column BPX70 (10 m × 0.1 mm i.d., 0.2 µm f.t.) from SGE Analytical Science (Melbourne, Australia). The injector and detector temperatures were set at 240 °C. Carrier gas (hydrogen) flow was 0.80 mL/min. The oven temperature was held at 50 °C for 0.2 min, increased from 50 to 175 °C at 120.0 °C/min, was held at 175 °C for 2 min, from 175 to 220 °C at 20.0 °C/min and finally from 220 to 250 °C at 50.0 °C min^−1^. The samples were injected in split mode (0.4 µL) with a split ratio set at 1:20. Peak identification was accomplished by comparing peak retention times with GLC-463, menhaden oil, and FAME 189-19 standard mixtures. EPA and DHA were quantified using a calibration curve of the respective standards. All the others were reported as g/100 g of fatty acids. To evaluate the precision of the method, the amount of EPA and DHA of FAME189-19 mix was calculated, reaching similar results as reported in the technical sheet. The intraday and interday repeatability were also valuated for EPA and DHA. The intraday repeatabilities (expressed as % RSDs) of the total peak area were from 0.56 and 089% for EPA and DHA, respectively, whereas the interday repeatabilities were 1.24 and 1.96%, respectively.

### 2.4. Triglycerides (TAG) Composition by GC-FID

Triglycerides were determined on supplement oils according to Guerra et al. [17] into a GC-FID from Shimadzu (GC-2010 Plus, Shimadzu Corporation, Kyoto, Japan); the separation was performed with a Rtx-65 TG fused silica capillary column (30 m, 0.25 mm i.d., 0.10 µm f.t.) with 35% dimethyl and 65% diphenyl polysiloxane from Restek (Chromatography Products, Superchrom, Milano, Italy). The initial oven temperature of 240 °C was raised to 370 °C at a rate of 2.5 °C min^−1^. The injector and detector temperatures were set at 360 °C. Hydrogen was used as a carrier gas at a flow rate of 1.75 mL min^−1^. The split ratio was set at 1:30. According to Fontecha et al. [18], TAG classes were separated according to the total number of carbon atoms and were expressed as percentage of total areas.

### 2.5. TLC Fractionation and GC–MS Analysis of Lipid Classes

To separate the lipid classes, a preparative TLC was conducted. Briefly, 20 mg of fat was dissolved in n-hexane and loaded on a TLC plate silica gel (20 cm × 20 cm; Merck, Darmstadt, Germany). Lipids were eluted using a mobile phase of n-hexane/diethyl ether (3:2 *v*/*v*). The lipid classes were identified using standard solutions of different lipids and in comparison with olive oil. An unidentified band was scraped off, extracted with chloroform, and dried with nitrogen. The residue was re-dissolved in n-hexane and injected in GC–MS. The analysis was conducted by a 6890 GC system coupled to an Agilent 5975A MS detector (Agilent Technologies, Santa Clara, CA, USA). A DB-5 column (60 m × 0.25 mm inner diameter, 0.25 µm film thickness) from Agilent Technologies was used for the separation. The oven was set at a temperature of 120 °C and then increased at a rate of 10 °C/min to 320 °C for 5. The injection was performed in split mode with a split ratio of 30:1, and the volume of injection was 1 μL. Helium was used as carrier gas at a rate of 1 mL/min. Mass source was ionized with electron-impact (EI) mode. Solvent delay: 1 min. Analyses were carried out in full scan mode at scan range of *m*/*z* 50–500.

### 2.6. Tocols Determination by HPLC-FLD

Tocols was carried out according to Gomez-Caravaca et al. [19]; α-tocopherol calibration curve was used to quantify them, assuming that the response of all tocols was the same as that for α-tocopherol (according to previous work of the research group). Briefly, the samples (1 g) were dissolved in 10 mL of n-hexane and analyzed in a HPLC (Agilent 1200) equipped with a fluorimeter detector. The excitation wavelength was 290 nm, and the emission wavelength was 325 nm. The tocols were separated in a Luna Hilic Phenomenex column (250 mm × 4.6 mm i.d., 5 µm particle size) in isocratic conditions at a flow rate of 0.5 mL/min. To identify the tocotrienols, a barley flour sample was saponified according to the procedure proposed by Panfili et al. [20], and the tocotrienols were identified by comparison between the barley sample and the supplements.

### 2.7. Determination of Peroxide Value (PV)

Peroxide value was determined according to the International Dairy Federation method proposed by Shantha and Decker [21]. Peroxides of supplement oils were reacted with Fe (II) and ammonium thiocyanate solutions, and the intensity of a red-violet complex was measured at 500 nm. PV was quantified according to a calibration curve of ferric chloride. In spite of this method is the official International Dairy Federation method for determination of the peroxide value of anhydrous milk; the same authors extended it to poultry, meat, fish, and vegetable oils. The results in most cases were consistent with those obtained by using the AOAC Official Method.

### 2.8. Determination of p-Anisidine Value (AV)

The AVs were determined according to the European Pharmacopoeia [22].

### 2.9. Calculation of Totox Value

According to Albert et al. [23], Totox was calculated as reported in Equation (1):

Totox = (2 × PV) + AV(1)

### 2.10. Statistical Analysis

The results reported are the averages of three replications, unless otherwise stated. Statistica 6.0 software (2001, StatSoft, Tulsa, OK, USA) was used to calculate one-way ANOVA (analysis of variance) couples with Tukey’s honest significant difference and to obtain the bloc plots. Before the ANOVA analysis, the data were normalized using the following formula:

zi = (xi − min(x))/(max(x) − min(x)) × 100(2)

where

zi: the ith normalized value in the dataset;
xi: the ith value in the dataset;
min(x): the minimum value in the dataset;
max(x): the maximum value in the dataset.

## 3. Results

### 3.1. Fatty Acid Composition of Omega-3-Supplements

**Figure 1:**
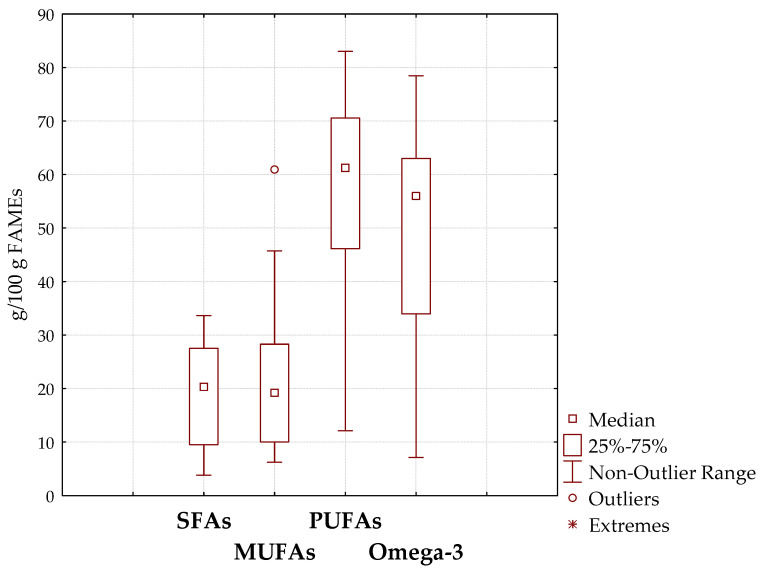
Total saturated (SFA), monounsaturated (MUFA), polyunsaturated (PUFA), and omega-3 fatty acid content in the analyzed supplements.

Fatty acid methyl-esters were analyzed by GC-FID, and a total of 37 fatty acids were determined. Figure 1 reports the distribution of fatty acid classes into analyzed supplements.

As expected, the analysis of FAME samples showed that PUFAs were the main fatty acids, with a mean of 58% of total fatty acids; however, their content ranged from 12.1 to 83% of total fatty acids. According to the PUFA trend, omega-3 fatty acids were in the range 7.1–78.5% with a mean of 48.8%. MUFAs were the second most abundant lipid classes with a mean of 22.2% and a range of 6.3–60.9%; however, all samples contained less than 50% of MUFAs except the S12 sample that reported 60.9% corresponding to a supplement of shark oil. This oil contains high amounts of squalene and alkylglycerols [24]; however, its content of omega-3 fatty acids was moderate. In fact, it showed the lowest omega-3 relative content (7.1%). Finally, SFAs accounted from 3.8 to 33.6% of total FAMEs with a mean of 19.8%.

Table 1 showed the omega-3 oil source and the content of total omega-3, EPA, and DHA for recommended serving; moreover, the label claim for serving was also described. Generally, the main supplements analyzed in this work met the label content of n-3 PUFA; however, some exceptions were noticed.

Generally, all samples exceed the label content by 3.8–39.5% for total omega-3 content, 1.4–58.3% for EPA content, and 3.5–68.8% for DHA content. Similar data were noticed by Nichols and co-workers [25] on Australian omega-3 supplements. Aside from the majority of the analyzed samples meeting the label claim, 2.5% of them did not meet the label content. Briefly, S2 and S5 total omega-3 contents were 8.8 and 21.3% lower than that declared, respectively; EPA contents in S6 and S8 samples were 153.2 and 131.3% lower than the declared claim, respectively; and finally, samples S1 and S10 showed DHA contents for servings that were 208.6 and 18.5% lower than the declared claim, respectively. Albert et al. [23] also reported that several New Zealand supplements had omega-3 concentrations noticeably lower than label claims.

The sum of EPA and DHA per serving was lower for half of the samples analyzed when compared with other countries such as the USA [26] and in agreement with German [27], Australian, and New Zealand [25] data, as well as in essence being higher than in the UK [28].

Table 2 reports the complete determination of the 37 fatty acids determined as methyl-esters.

As reported in Figure 1 and Table 2, a high dispersion of the data was reported, and they were dependent on the oils used in the formulations.

In fact, samples S1 and S2 showed high percentages (compared to the others) of linoleic and γ-linolenic acids, confirming the presence of borage oil [29].

Sample S7 was formulated, a part of *Schizochytrium* oil, with a mix of canola, cameline, and hemp oils; this confirms the high percentage of oleic acid and moderate amounts of linoleic and linolenic acids.

Samples S8 and S11 reported high amounts of linolenic acid (omega-3). This is justified because, as reported in the labels, in the first case, it was a mix of fish and vegetable linolenic oils; in the second case, the supplement was formulated with linseed oil.

Sample S12, formulated with shark liver oil, reported high amounts of monounsaturated fatty acids; according to the data shown by Bakes and Nichols [30], the sums of C16:1, Cl8:l, C20:1, C22:1, and C24:1 were higher than 70%.

In terms of EPA and DHA, samples S7 and S19 that contained *Schizochytrium* oil, as expected, reported very high amounts of DHA compared to EPA. Surprisingly, samples S14, S15, S17, and S18 that also contain *Schizochytrium* oil, a source of DHA fatty acid [31], also showed a high percentage of EPA ranging between 17.6 and 20.2%. However, some modified *Schizochytrium* algae are able to also produce EPA, and the high presence of this fatty acid was reported in the labels.

Analyzing the principal fatty acid classes (saturated, monounsaturated, and polyunsaturated fatty acids), a high variability was noticed; in fact, total SFA ranged between 3.8 and 33.6%, total MUFA varied between 5.8 and 60.9%, and PUFAs were in the range of 12.1–83.0%; thus, statistically significant differences in terms of total SFA, MUFA, and PUFA content was found among the supplements (*p* < 0.05). These data partially agree with the results showed by Karsli [32] that analyzed Turkish fish oil supplements.

### 3.2. Triglycerides Composition of Supplement Oils

On the basis of a GC-FID analysis, it was possible to separate tryglycerides from the other compounds (hydrocarbons, monoglycerides, diglycerides, free fatty acids, and phospholipids, among others).

**Figure 2:**
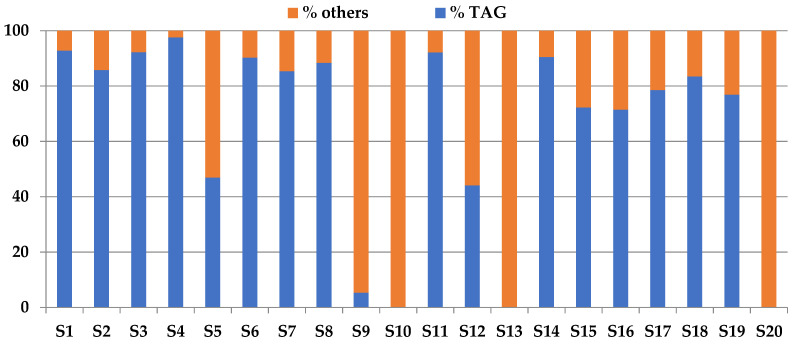
Relative content (%) of triglycerides (TAG) and other lipids in the samples (*n* = 3).

Figure 2 shows the distribution between these groups of compounds.

The TAG range content was 0–97.6%. Samples S10, S13, and S20 did not contain TAG because they were formulated with concentrated fish oil containing ethyl esters of EPA and DHA. Sample S5 showed a TAG content of 47%; this low content was strictly related to the source of the oil. In fact, this sample was formulated with krill oil; according to the study carried out by Castro-Gómez et al. [33], TAG represent the second class of total lipids just after the phospholipids and followed by DAGs. Samples S12 that contained shark liver oil also showed a low TAG content (44%); these data agreed with the results shown by Bakes and Nichols [30] that noticed that TAG content in the liver oil of different sharks ranged between 5 and 49.1% and other mayor compounds were diglyceride-ethers (9.9–76.6%) and hydrocarbons as squalene (0–81.6%).

Samples containing microalgae oils (S7, S14, S15, S16, S17, S18, and S19) showed high variability of TAG content from 71.4 to 90.5%; in spite of TAG being the main lipid class in Schizochytrium oil, as reported by Wand and Wang [34], the content of free fatty acids and polar lipids is variable, and moreover the distribution between neutral and polar lipids depends on the growing time [35].

Finally, sample S9 formulated with fish oil reported a very low content of TAG (5.3%). To confirm the data obtained by GC-FID, lipid classes were separated by TLC.

**Figure 3:**
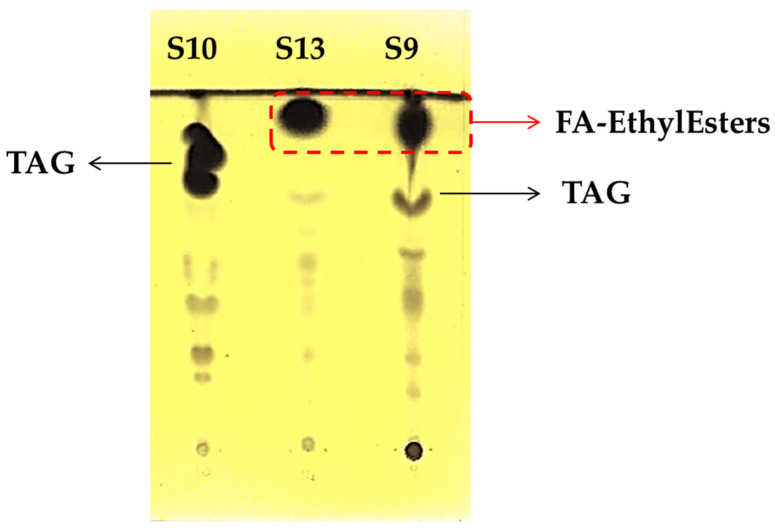
TLC separation of lipid classes in S9, S10, and S13 samples.

The S9 samples showed two main spots at the end of elution; according to the comparison with other samples, the first one (lower Rf) is related to the triglycerides, and the last one (higher Rf) presented the same elution of the ethyl esters present in the fish oil concentrates (Figure 3). To confirm the identity, the spot was scraped off and analyzed by GC–MS, confirming the presence of ethyl esters of omega-3 fatty acids. Briefly, the fragment at *m*/*z* 108, typical of omega-3 fatty acids, was detected. Moreover, the fragments at *m*/*z* 79, 91, and 119 were found for EPA ethyl ester, and the fragments at *m*/*z* 79, 91, and 117 were detected for DHA ethyl ester.

According to this information, we suppose that these samples were formulated with concentrate fish oil, despite the fact that it was not reported on the label. This hypothesis was also confirmed by the fatty acid composition (Table 2); in fact, the sum of EPA and DHA was higher than 53%.

Triglyceride classes were identified according to Fontecha and co-workers [18]. Analysis by GC-FID permitted the identification of 13 CN (carbon number) groups (from CN34 to CN58); the analysis showed that short-chain TAG group (SC-TAG) (CN26-CN40) was poorly represented (Table 3), except in sample S9 that reported the 39% of total TAG.

Long-chain TAG (LC-TAG) from CN48 to CN58 was the main triglyceride class, ranging from 57 to 100% of total triglycerides, except in sample S9, where they represented only 9% of total TAG. In other words, medium-chain TAG (MC-TAG) from CN42 to CN46 ranged from 0 to 37%, except in sample S9 that contain 53% of this class.

Literature on TAG composition of different oil is scarce, and the lack of information on the ratio of different oils when they were mixed makes it hard to discuss the data. However, analyzing sample S5 with krill oil, we obtained similar results to those reported by Castro-Gómez and co-workers [33]; in fact, also in our sample, CN48 represented the first TAG class (40%), followed by CN46 (25%) and CN50 (16%).

The data obtained for fish oils and fish oil concentrates regarding TAG percentage were in line with the results of Sprague et al. [28] in terms of UK fish oil products.

### 3.3. Tocols Composition of Supplement Oils

**Figure 4:**
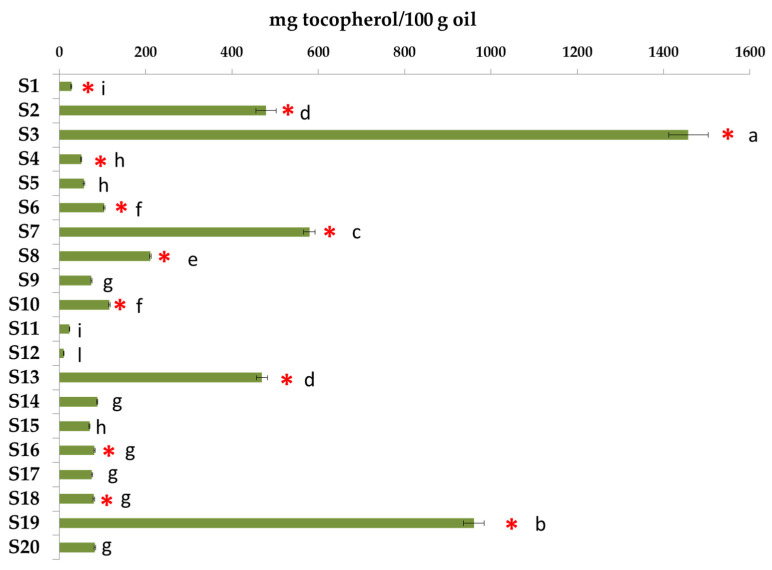
Total content of tocols (mg/100 g oil). Red asterisk indicates the addition of tocopherols as ingredients as reported in the label. Error bars present the standards deviation (*n* = 3). Different letters report statistical differences (*p* < 0.05).

Tocols of the supplement samples were determined by HPLC-FLD. A total of six tocols were identified: four of them were tocopherols (α-, β-, δ-, and γ-tocopherol) and two were tocotrienols (α- and β-tocotrienol). Figure 4 shows the total content of tocols in the samples.

**Figure 5:**
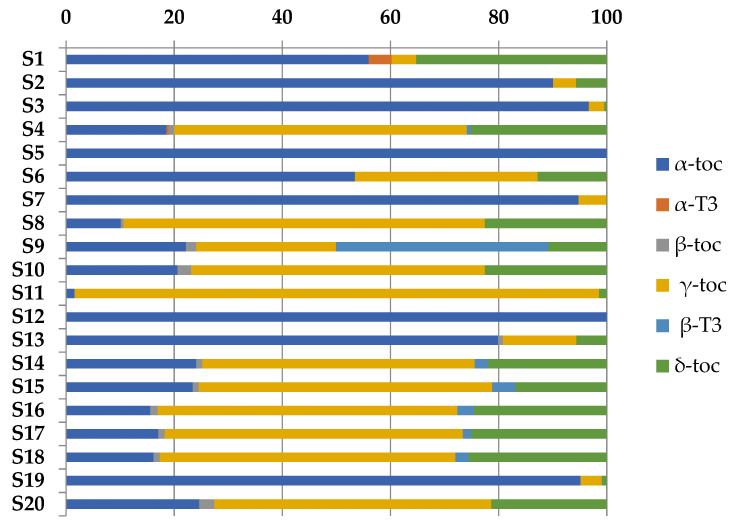
Tocol composition (%) of supplements (*n* = 3).

Figure 5 reports the distribution of the different tocols in the supplements.

Tocol content was very different among the samples; in fact, the total amounts varied from 10.1 to 1457.2 mg/100 g of oil. Any relation was found between the tocol content and the addition of vitamin E as declared on the label. α-Tocopherol was the first tocol in S1, S2, S3, S6, S7, S12, S13, and S20; otherwise, samples S4, S8, S10, S11,S14, S15, S16, S17, S18, and S20 contained γ-tocopherol as the main one. In particular, sample S11, formulated with linseed oil, reported 97% of this compound. The presence of tocotrienols was due to the mix of marine/algae oil with other vegetable oils.

### 3.4. Oxidative Status of Supplement Oils

To establish the lipid quality of the supplements, the oxidative status was also determined, valuating the peroxide value (PV), *p*-anisidine value (*p*-AV), and Totox according to the Global Organisation for EPA and DHA (GOED) guidelines [36].

**Figure 6:**
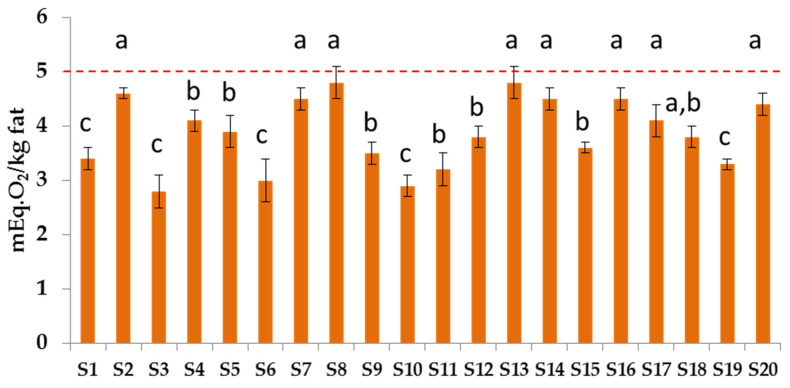
Peroxide values (PV) of oil of supplements (red dotted line indicates the GOED maximum limit of 5 mEq. O_2_/kg). Error bars present the standard deviation (*n* = 3). Different letters report on the statistical differences (*p* < 0.05).

PV, a marker of the first stage of oxidation, is reported in Figure 6 for all the samples. The average of PV values obtained in the samples was 3.9 ± 0.6 mEq. O_2_/kg of oil, and the single values ranged between 2.8 and 4.8 mEq. O_2_/kg of oil. All the supplements under study met the GOED guidelines, showing a PV < 5; however, six of them reported PV > 4.5.

**Figure 7:**
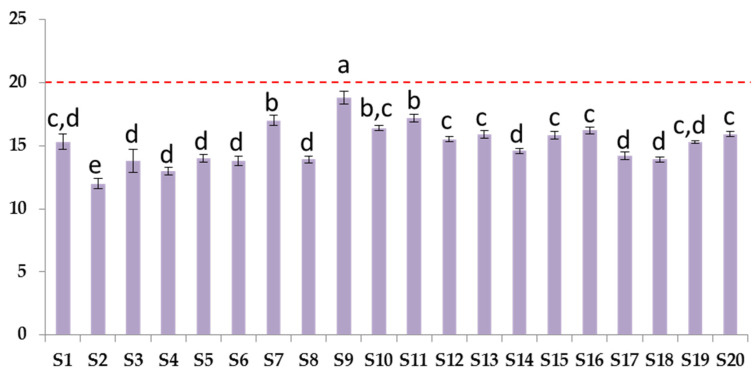
*p*-Anisidine values of supplements (red dotted line indicates the GOED maximum limit of 20). Error bars present the standard deviation (*n* = 3). Different letters report the statistical differences (*p* < 0.05).

*p*-Anisidine (*p*AV) was used as a marker of secondary oxidation stage (Figure 7).

The average of *p*-anisidine value was 15.1 ± 1.6, and minimum and maximum were 12 and 18.8, respectively. Moreover, in this case, all samples complied with the GOED recommended levels.

Furthermore, we know that colored oils such as krill oil cannot be tested for *p*AV value [37] due to the interference of color that could generate false positives. We can affirm that this sample also showed a low *p*AV value.

To corroborate if good practices were performed in the lab, the ratio PV/*p*AV was calculated; in fact, according to the suggestion of Bannenberg et al. [37], if this ratio is higher than 1, it means that the samples suffered an oxidation during their manipulation in the lab. The results obtained in our work noticed a ratio in a range of 0.2–0.4, confirming the optimal manipulation of the samples before the analyses.

**Figure 8:**
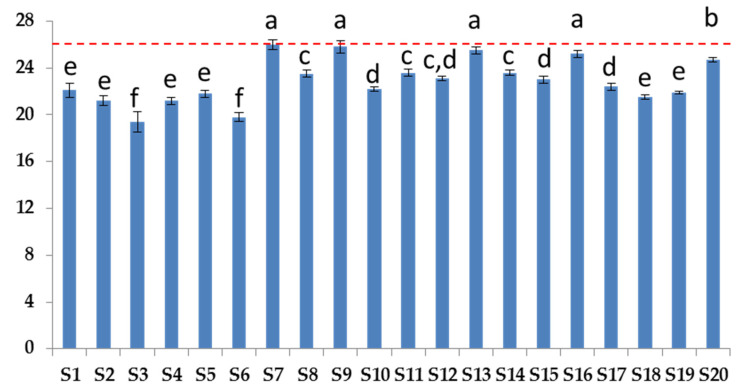
Totox values of supplements (red dotted line indicates the GOED maximum limit of 26). Different letters report statistical differences (*p* < 0.05).

PV and *p*AV values are usually used to calculate the Totox. Figure 8 reports the Totox values for all samples.

As shown in Figure 8, there were no samples above the recommended GOED level; the average Totox number was 22.9 ± 1.9, and 100% of the products complied with the maximum limit of 26 provided by GOED for fish oil. The samples showed Totox value in the range of 19.4–25.9.

The low levels of oxidation identified in this study were consistent with other recent studies. In alignment with Nichols et al. [25] who confirmed that the Australian and New Zealand fish oil are not oxidized, French products also showed the same trend. However, additional analyses such as the determination of volatile compounds (i.e., aldehydes) are necessary to confirm the low lipid oxidation.

## 4. Conclusions

In sum, omega-3 supplements containing fish, krill, or microalgal oil were analyzed in terms of lipid composition and for lipid oxidation. Only 2.5% of the analyzed samples did not meet the label content of omega-3 fatty acids. Analysis of FAMEs and TAGs also showed that one sample contained omega-3 ethyl esters, but it was not reflected on the label. TAG analysis showed high variability among the triglyceride classes; this depended on the source of omega-3 and on the composition of the other oils that sometimes were added during the cap formulation. We also determined the tocol content, and a high variability of the distribution of the six tocols (four tocopherols and two tocotrienols) was found among the samples. The amounts of tocols contained in the samples were not strictly dependent on the addition of vitamin E in the supplements. Finally, lipid oxidation analyses (PV, pAV, and Totox) confirmed that the analyzed samples met the GOED requirement and could be considered safe for the consumer.

## Figures and Tables

**Table 1:** Total omega-3, EPA, and DHA content (mg/serving) in the omega-3 supplements and comparison with the amounts declared in the label.

Suppl. Nº	Oil/Omega-3 Source	Serving (Caps)	Total Omega-3 mg/Serving	Total EPA mg/Serving	Total DHA mg/Serving	Labelled Content/Serving
S1	Fish and borage oils	2	230.5	201.3	16.2	• Omega-3: 174 mg • EPA: 84 mg • DHA: 50 mg
S2	Fish and borage oils	2	147.0	85.5	45.2	• Omega-3: 160 mg
S3	Fish oil	2	757.7	438.9	346.3	• EPA: 420 mg • DHA: 280 mg
S4	Fish oil	3	519.6	292.4	174.0	• Omega-3: 500 mg • EPA: 240 mg • DHA: 160 mg
S5	Krill oil	2	259.6	177.6	73.2	• Omega-3: 315 mg • EPA + DHA: 250 mg
S6	Cod liver oil	3	446.7	257.1	157.8	• EPA: 651 mg • DHA: 135 mg
S7	Canola, hemp, Camelina sativa, Schizochytrium oils	2	569.2	30.5	231.5	• DHA: 200 mg
S8	Fish and vegetable oils	2	1511.4	129.7	320.4	• EPA: 300 mg • DHA: 100 mg
S9	Fish oil	2	633	330	239.6	• EPA: 330 mg • DHA: 220 mg
S10	Fish oil concentrate	2	752.2	119.9	422.0	• Omega-3: 666 mg • EPA: 108 mg • DHA: 500 mg
S11	Linseed oil	1	433.3	0.2	0.8	• ALA: 285 mg
S12	Shark liver oil	2–4	34.4–68.9	27.2–54.3	3.8–7.5	• Omega-3: 60 mg
S13	Fish oil concentrate	3	2089.5	1065.3	833.1	• EPA: 1050 mg • DHA: 750 mg
S14	Schizochytrium oil	2	648.1	233.2	369.7	• EPA: 167 mg • DHA: 333 mg
S15	Schizochytrium oil	2	664.5	219.5	417.6	• EPA: 200 mg • DHA: 400 mg
S16	Microalgae and vegetable oils	2	1380.0	371.8	518.2	• Omega-3: 835 mg • EPA: 300 mg • DHA: 500 mg
S17	Schizochytrium and vegetable oils	2	571.3	183.6	350.9	• EPA: 125 mg • DHA: 250 mg
S18	Schizochytrium and vegetable oils	1	548.0	168.9	360.2	• EPA: 125 mg • DHA: 250 mg
S19	Schizochytrium oil	1	301.4	8.3	289.3	• DHA: 250 mg
S20	Anchovy oil concentrate	1–3	368.0–1103.9	194.2–582.5	143.0–428.9	• EPA: 180–540 mg • DHA: 120–360 mg

**Table 2:** Fatty acid content (g/100 g FAMEs) of omega-3 supplements (*n* = 3).

FAMEs	S1	S2	S3	S4	S5	S6	S7	S8	S9	S10	S11	S12	S13	S14	S15	S16	S17	S18	S19	S20
**C6:0**	0.02 d	0.02 d	0.02 d	0.10 b	0.07 c	0.02 d	0.02 d	0.02 d	0.10 b,	0.41 a	0.02 d	0.14 b	0.02 d	0.02 d	0.01 e	0.01 e	0.02 d	0.01 e	0.01 e	0.03 d
**C8:0**	0.18 e	0.33 b	0.28 c	0.45 a	-	0.33 b	0.18 e	0.21 d	0.42 a	0.31 b	0.23 d	0.18 e	0.15 e	0.14 e	0.22 d	0.37 b	0.42 a	0.28 c	0.15 e	0.01 f
**C10:0**	0.13 d	0.26 s	0.19 d	0.32 a	-	0.15 d	0.14 d	0.16 d	0.32 a	0.24 b	0.17 d	0.17 d	0.10 e	0.10 e	0.16 d	0.25 b	0.29 a	0.21 c	0.11 e	0.01 f
**C11:0**	0.001 b	-	0.002 b	0.02 a	-	-	0.0004 b	0.0004 b	0.01 a	0.003 b	-	-	-	0.003 b	0.003 b	0.001 b	0.002 b	0.002 b	0.003 b	0.01 a
**C12:0**	0.06 b	0.04 b	0.002 d	0.11 a	0.13 a	0.03 b	0.04 b	0.002 d	0.02 b	0.003 d	0.003 d	0.06 b	0.01 c	0.09 a	0.07 a	0.10 a	0.10 a	0.07 a	0.08 a	0.01 c
**C12:1c**	0.01 b	0.004 c	-	0.02 b	0.01 b	0.003 c	0.01 b	-	-	0.004 c	-	0.01 b	1.18 a	0.002 c	0.002 c	0.002 c	0.002 c	0.001 c	-	-
**C14:0**	4.16 b	2.95 d	0.14 f	7.07 a	7.52 a	4.08 b	1.32 e	0.12 f	0.19 f	0.03 g	0.04 g	2.60 d	0.28	1.47 e	1.19 e	1.54 e	1.66 e	1.16 e	3.64 c	0.16 f
**C14:1c**	0.22 c	0.10 c	0.01 d	0.23 c	0.31 b	0.29 b	0.02 d	0.01 d	0.01 d	0.01 d	-	0.42 a	0.03 d	0.01 d	0.01 d	0.01 d	0.01 d	0.01 d	0.08 c	0.02 d
**C15:0**	0.16 d	0.24 c	0.01 f	0.49 b	0.39 b	0.29 c	0.08 e	0.02 f	0.02 f	0.003 g	0.02 f	0.42 b	0.03 f	0.82 a	0.28 c	0.42 b	0.33 b,c	0.29 c	0.22 d	0.02 f
**C15:1c**	0.06 b	0.06 b	0.004 d	0.10 a	0.15 a	0.12 a	0.02 c	0.01 c	0.01 c	0.002 d	-	0.16 a	0.01 c	0.02 c	0.02 c	0.03 c	0.03 c	0.02 c	0.05 b	0.004 d
**C16:0**	9.46 f	12.60 e	0.89 m	15.87 d	22.51 b	9.46 f	7.73 g	3.72 i	5.92 h	0.06	4.92 h	20.03 c	1.38 l	15.77 d	19.28 c	26.54 a	18.88 c	21.23 b	11.73 e	1.38 l
**C16:1t**	0.20 d	0.23 d	0.03 e	0.60 a	0.34 c	0.43 b	0.03 e	0.02 f	0.03 e	0.01 f	0.02 f	0.60 a	0.05 e	-	-	-	-	-	-	0.03 e
**C16:1c**	5.78 c	4.53 c	0.52 e	8.63 a	9.53 a	8.28 a	0.17 f	0.18 f	0.62 e	0.29 f	0.06 h	7.33 b	1.34 d	0.09 g	0.08 g	0.10 g	0.05 h	0.06 h	0.16 f	1.02 d
**C17:0**	0.09 d	0.31 c	0.13 d	0.48 b	1.17 a	0.16 d	0.07 e	0.06 e	0.14 d	0.09 d	0.05 e	0.38 b	0.12 d	0.42 b	0.05 e	0.09 d	0.10 d	0.07 e	0.09 d	0.25 c
**C17:1**	0.09 d	0.09 d	0.05 e	0.21 c	0.15 c	0.31 b	0.03 e	0.03 e	0.10 d	0.01 f	0.03 e	0.82 a	0.10 d	0.01 f	0.01 f	0.01 f	0.01 f	0.01 f	0.02 f	0.15 c
**C18:0**	2.23 c	3.67 b	3.35 b	3.35 b	1.01 d	2.02 c	1.81 d	2.05 c	3.31 b	0.05	3.37 b	2.57 c	2.09 c	1.79 d	1.79 d	1.97 d	1.52 d	1.73 d	0.67 e	4.94 a
**C18:1t**	0.36 c	1.17 b	0.18 d	2.81 a	0.83 b	0.38 c	-	0.05 e	0.20 d	0.08 e	-	0.71 b	-	-	-	-	-	-	-	0.20 d
**C18:1c9**	15.18 e	15.54 e	9.90 f	10.69 f	12.39 e	21.88 d	31.02 b	8.63 f	10.70 f	0.30 h	18.21 d	39.61 a	7.04 g	24.15 c	9.72 f	6.64 g	5.91 g	8.45 f	8.20 f	13.46 e
**C18:2tt**	0.27 c	0.05 e	0.11 d	0.11 d	0.14 d	0.45 b	-	0.01 f	0.13 d	0.03	-	0.24 c	0.20 c	-	-	-	-	-	-	3.23 a
**C18:2n6**	15.12 c	21.72 a	1.52 e	1.40 e	1.75 e	1.69 e	17.49 b	9.24 d	2.37 e	0.08 f	14.89 c	0.93 e	0.96 e	1.79 e	0.61 f	0.72 f	0.79 f	0.96 e	1.17 e	1.39 e
**C18:3n6**	8.66 a	9.72 a	0.12 e	0.52 b	0.08 f	0.22 d	0.34 c	0.06 f	0.41 b	0.03 g	0.15 e	0.12 e	0.31 c	0.01 g	0.02 g	0.01 g	0.01 g	0.01 g	0.01 g	0.45 b
**C19:1**	-	-	0.25 b	0.34 a	0.33 a	0.21 c	0.06 e	0.21 c	0.19 c	0.01 e	0.12 d	0.36 a	0.29 a	0.02 e	0.04 e	0.02 e	0.04 e	0.03	0.26 b	0.40 a
***C18:3n3***	*0.38 g*	*0.99 e*	*0.54 f*	*0.85 e*	*0.62 f*	*0.76 f*	*14.03 c*	*48.87 b*	*2.20 d*	*0.02 l*	*56.96 a*	*0.23 h*	*0.66 f*	*0.09 i*	*0.04 l*	*0.05 l*	*0.06 l*	*0.05 l*	*0.05 l*	*1.05 e*
**C20:0**	0.16 g	0.13 g	2.13 a	0.23 f	0.05 h	2.08 a	0.76 c	0.13 g	1.29 b	0.10 g	0.12 g	0.15 g	0.68 c	0.34 e	0.50 d	0.63 c	0.39 e	0.47 d	0.08 g,h	1.05 b
**C20:1**	14.39 a	5.13 c	3.70 d	5.05 c	2.59 e	13.43 a	5.08 c	0.49 f	3.89 d	0.59 f	0.16 g	9.47 b	3.99 d	0.10 g	0.11 g	0.12 g	0.08 g	0.10 g	0.04 h	4.43 c
**C20:2n6**	0.20 c	0.19 c	0.38 b	0.20 c	0.10 d	0.28 b	0.57 a	0.08 d	0.31 b	0.03 e	0.03 e	0.28 b	0.20 c	0.03 e	0.02 e	0.02 e	0.02 e	0.02 e	0.01 e	0.40 b
**C20:3n6**	0.04 d	0.13 c	0.52 a	0.25 b	0.14 c	0.13 c	0.11 c	0.08 d	0.35 a	0.09 c	-	0.12 c	0.38 a	0.05 d	0.06 d	0.09 c	0.08 c	0.06 d	0.40 a	0.47 a
**C20:4n6**	0.11 e	0.48 d	2.15 a	1.19 c	0.47 d	0.37 d	0.12 e	0.37 d	1.75 b	0.45 d	-	0.18 e	2.38 a	1.27 c	1.42 c	1.55 c	1.43 c	1.31 c	0.47 d	1.81 b
***C20:3n3***	*0.03 e*	*0.06 d*	*0.32 a*	*0.10 d*	*0.05 d*	*0.13 c*	*0.37 a*	*0.08 d*	*0.20 b*	*0.05 d*	*0.05 d*	*0.17 c*	*0.21 b*	*0.16 c*	*0.17 c*	*0.21 b*	*0.23 b*	*0.20 b*	*0.06 d*	*0.38 a*
**C22:0**	0.28 e	0.57 d	1.82 a	1.02 b	0.73 c	0.76 c	0.23 e	0.12 f	2.15 a	0.42 d	0.10 f	0.18 e,f	2.00 a	0.88 b	0.89 b	1.03 b	0.91 b	0.96 b	0.90 b	1.90 a
***C20:5n3***	*18.98 f*	*10.75 g*	*34.79 b*	*21.0 6*	*25.15 d*	*17.94 f*	*1.47 i*	*6.27 h*	*31.20 c*	*12.50 g*	*0.03 l*	*5.62 h*	*37.22 a*	*17.57*	*20.22 e*	*21.69 e*	*20.65 e*	*18.20 f*	*1.09 i*	*32.60 c*
**C22:2**	0.06 c	0.04 d	0.17 b	0.04 d	0.02 e	0.07 c	0.16 b	0.04 d	0.10 c	0.09 c	0.02 e	3.01 a	0.11 c	0.04 d	0.02 e	0.01 e	0.02 e	0.02 e	0.002 f	0.14 b
**C22:3 + C22:4**	0.01 h	0.05 g	1.18 c	0.11 f	0.04	0.49 e	0.32 e	0.10 f	0.67 d	1.24 c	0.02 h	0.09 f	1.32 c	1.30 c	1.61 c	2.27 b	2.37 b	1.57 c	15.81 a	0.69 d
**C24:0**	0.05 h	0.20 h	0.24 h	0.47 g	0.05 h	-	4.60 b	0.62 f	3.60 c	2.10 d	0.08 h	0.07	0.14 i	0.27 h	0.21 h	0.25 h	0.18 h	1.57 e	15.81 a	0.08 l
**C24:1**	1.05 c	0.96 c	5.38 b	0.10 g	0.28 f	0.40 e	0.02 i	0.11 g	0.82 d	14.34 a	0.01 i	1.43 c	0.13 g	0.17 f	0.38 e	0.37 e	0.11 g	0.27 f	0.05 h	0.09 g
***C22:5n3***	*0.29 l*	*0.97 g*	*27.45 b*	*2.88 e*	*0.56 h*	*1.33 f*	*0.40 i*	*2.32 e*	*3.60 d*	*44.03 a*	*0.01 m*	*0.32 i*	*5.80 c*	*3.16 d*	*2.33 e*	*2.65 e*	*3.84 d*	*1.79 f*	*0.39 i*	*3.75 d*
***C22:6n3***	*1.53 i*	*5.69 h*	*1.52 i*	*12.54 f*	*10.36 g*	*11.01 g*	*11.16 g*	*15.49 e*	*22.65 d*	*21.87 d*	*0.11 m*	*0.80 l*	*29.11 b*	*27.85 b*	*38.47 a*	*30.23 b*	*39.45 a*	*38.81 a*	*38.18 a*	*24.00 c*

Omega-3 fatty acids are reported in italics. Different letters in the same line show statistical differences (*p* < 0.05).

**Table 3:** Triglyceride composition (on total lipids) of supplement samples (*n* = 3).

	S1	S2	S3	S4	S5	S6	S7	S8	S9	S10	S11	S12	S13	S14	S15	S16	S17	S18	S19	S20
**T34**					0.4 a	0.1 b			0.3 a											
**T36**					0.8 a	0.3 c		0.5 b	0.6 b											
**T38**					0.3 d	0.8 b		2.4 a	0.5 c			0.8 b								
**T40**			0.7		1.0 d	1.1 d	0.4 e,f	1.5 b	0.6 e		1.3 c	2.0 a								
**T42**			0.5 f		2.4 c	1.3 d	2.3 c	7.7 a	1.1 e		6.3 b	0.2 g								
**T44**			2.4 c	3.1 a	3.3 a	0.8 e	0.3 f	2.9 a,b	1.1 d			1.0 d								
**T46**	2.8 f	4.5 e	4.9 d	20.1 a	11.6 b	5.6 c		1.6 h	0.6 i			2.5 g								
**T48**	7.8 d	7.1 e	6.1 f	27.3 a	19.1 b	15.3 c		0.8 g	0.3 h			7.1 e								
**T50**	14.1 d	10.6 e	13.8 d	19.1 c	7.5 f	24.1 a	2.8 h	2.6 i	0.2 l		3.2 g	20.6 b						10.6 e		
**T52**	29.5 a,b	30.6 a	29.7 a	7.7 h	0.5 n	23.0 c	23.4 c	10.4 f			16.9 d	6.4 l		14.2 e	9.9 g	9.7 g	6.9 i	6.3 l	4.9 m	
**T54**	19.1 g	15.9 l	18.6 h	16.6 i		12.8 l	46.6 e	22.3 f			21.9 f	3.0 m		76.2 a	62.3 d	61.8 d	71.7 b	66.5 c	72.0 b	
**T56**	17.6 c	17.1 d	14.2 e	3.8 h		5.1 g	8.1 f	35.8 b			42.5 a	0.5 i								
**T58**	1.9 a		1.3 c				1.5 b													
**SC-TAG**			0.7 e		2.5 b	2.3 b,c	0.3 f	4.4 a	2.1 c		1.3 d	2.8 b								
**MC-TAG**	2.8 h	4.5 f	7.8 d	23.1 a	17.3 b	7.7 d	2.6 h	12.2 c	2.8 h		6.3 e	3.7 g								
**LC-TAG**	90.0 a	81.3 c,d	83.7 b	74.5 g	27.1 i	80.3 d	82.4 c	71.8 h	0.5 l		84.5 b	37.6		90.5 a	72.2 h	71.4 h	78.6 e	83.5 b	76.9 f	

Abbreviations are as follows: SC-TAG, short-chain triglyceride; MC-TAG, medium-chain triglyceride; LC-TAG, long-chain triglyceride. Different letters in the same line showed statistical differences (*p* < 0.05).

## Data Availability

Not applicable.
